# On Puerperal Irritability

**Published:** 1825-02

**Authors:** C. Waller


### Art. III.—*On Puerperal Irritability.* By C. Waller, Esq.

Of all the diseases which, in a professional capacity, we are called upon to witness, there are none so rapid in their course, and often so fatal in their result, as those of the puerperal state; and perhaps none, the treatment of which is so undecided. The term *puerperal fever* is applied to such various states and conditions of the body, that we cannot wonder at the very opposite modes of treatment which have been recommended. Thus, we find one man relying principally, if not solely, on the use of the lancet, conjoined with purgatives and other remedies of a decidedly antiphlogistic character; a second considers it to be of the nature of putrid fever, and exhibits bark and acids; whilst a third tells you it is to be cured by external fomentations, and each brings forward proofs of success. The fact is, that simple peritonitis, occurring in the puerperal state, is often confounded with true puerperal fever; and hence it is that many patients are cured by large and repeated bleedings, for this practice we all know is the proper one in acute inflammation of the peritoneum, more especially if followed by a large dose of opium. I have been repeatedly gratified in observing the beneficial effect of the opiate after venesection, in keeping down that state of irritability so consequent upon the use of the lancet. Nothing, indeed, but a succession of facts could have rooted out the strong prejudice I had imbibed against the use of opium in every species of inflammatory affection.

With regard to the true puerperal fever, so far as my observation extends, the use of the lancet is hazardous, to say the least of it. Its essential characteristic is *excessive action* with *diminished power*, and I have several times seen the most decidedly bad effects follow the abstraction of blood; it appearing to extinguish what little power was left in the system. Dr. James Curry, in his Lectures on the Practice of Medicine, justly observes, that "action may take place when power is rapidly diminishing, and generally, the more rapid the inflammation, the less is there of power, as in erysipelas; and, when this takes place in large towns, we are obliged to overlook, in the treatment, the increased action of the vessels, and at once to give those remedies that support the system."* I shall not, however, at this time offer any further observations on this head, as it is to another form of puerperal disease that I wish to direct the attention of the profession.

Every one who has been much engaged in midwifery practice, must have observed that there are two species of disease, equally dangerous in their nature, and to which the puerperal

* MSS. Lectures, 1818.

female is obnoxious; in the one the abdomen is affected, in the other the head (and this totally distinct from puerperal mania). This affection appears most frequently to take place after a copious loss of blood, though I have known it occur, and to be attended with a fatal result, where there has not been the slightest hemorrhage. This patient was a young female of great delicacy, in whom the uterine efforts were very feeble: in fact, she had not a single good pain from the commencement to the termination of her labour, and I thought it right at last to expedite the delivery with the forceps. The child was born alive, and she appeared to be doing well. At the expiration of thirty-six hours, I found her in a state of extreme restlessness; she had a sensation of weight in the abdomen, but no pain, even on the application of a considerable degree of pressure; her pulse was 140, small and fluttering; her tongue white. On asking her how she did? she replied, with great earnestness and sharpness of voice, that she was very well. Now it is to this hurried, quick mode of speaking, that I wish most especially to direct the attention, as I conceive it to be one of the peculiar characteristics of the disease. It was this state, conjoined with the frequency of pulse (a symptom to be dreaded in every form of puerperal disease), that alarmed me, and I at once gave a prognosis, that was received with a great degree of disappointment by the patient's friends; for so well did she seem on the preceding evening, that her mother, who nursed her, had undressed herself and had got into bed. Her bowels had not been opened since her delivery; my intentions were, therefore, to give a purgative in the first instance, and afterwards to give opium freely. A period of twenty-four hours elapsed before a cathartic effect was produced, and then a profuse, liquid, involuntary, and highly offensive, discharge took place from the bowels, which continued till her death. The symptoms of cerebral disturbance increased, delirium supervened, though she could, without much difficulty, be roused so as to give a rational answer to any question that was proposed to her. In about forty-eight hours from the commencement of the attack, she died. I regret greatly to state that the head was not examined; the abdomen was very carefully, and no traces of recent disease were found. A quantity of very pale-coloured serum was effused into the abdominal cavity, and every part appeared whiter than natural. The left ovarium was converted into a cyst, containing that peculiar cheesy substance so often met with in scrofulous abscesses, and a quantity of hair. One of the glands of the mesentery also contained the same sort of matter mixed with hair.

I had an opportunity, a short time since, of witnessing another fatal case of this nature, but here the patient survived till the ninth day after delivery. She had lost a very considerable quantity of blood before delivery, in consequence of the implantation of the placenta over the mouth of the womb ; this organ contracted well after the child was delivered, and for two days the patient appeared to be doing well, though the pulse was all along in an accelerated state, varying, however, from 110 to 130 in the minute. About this period she had a decided chill, followed by increased heat, and afterwards a very free, warm, and equable perspiration. This latter symptom, however, gave no relief; for, in this perspirable state, the pulse kept up, and cerebral irritation increased ; she was quite delirious. Due attention was paid to the bowels, and opium administered in doses of a fluid drachm of the tincture every four hours; and this practice was attended with the most marked diminution of the morbid symptoms; the patient slept well, and awoke refreshed. Still, with all these flattering appearances, the pulse continued in an accelerated state, and she had frequent relapses. On the eighth day after delivery, she had an attack of mucous diarrhœa, and on the ninth she expired.

On examination of the abdomen, no disease was found there, excepting an enlargement of some of the mucous glands of the large intestines. The uterus was well contracted, and, as well as its appendages, was perfectly healthy. Considerably increased vascularity was observed in the brain; its substance, when cut into, exhibited a great number of bloody points, though not so many as I have observed in the examination of patients who have died of fever. A small quantity of milky serum was effused between the tunica arachnoides and pia mater; and the adhesion of the dura mater to the skull was so great, as to render their separation excessively difficult. Her age was about thirty-six.

For this case I am indebted to Mr. Cox, of the Broadway, in whose practice it occurred, and who politely invited me to visit it with him.

I have also had another opportunity of observing the effects produced upon the brain in consequence of hemorrhage, in a patient to whom I was called a short time since, and found the placenta, in this case also, over the mouth of the womb. This poor creature had lost a very large quantity of blood previous to my arrival; a considerable quantity also flowed during the delivery of the child, which so exhausted her that she died in three hours afterwards, during which time she was delirious. A full dose of opium was given, but the delirium had commenced previous to its exhibition; it could not, therefore, be attributed to that remedy.

Having detailed these unsuccessful cases, it is now my pleasing task to record one where the result was favourable, though the symptoms were extremely alarming. The subject of this case was a lady, ætatis thirty-seven, delivered of her first child after a short and natural labour, on the 25th of last month (October). The placenta was retained, and, a very considerable hemorrhage coming on, I judged it right to remove it, and in so doing found it to be adherent throughout the greater part of its extent. A very alarming state of syncope supervened previous to its removal, and continued for several hours afterwards, although there was but little after-hemorrhage. She complained of intense pain at the precordia, which was relieved by administering a dose of laudanum in some warm gruel, to which a little essence of ginger had been added. She at length rallied, and appeared to be going on favourably till the 29th; with the exception, however, of weight and pain in the head, to which she had been subject for some time previous to her confinement. Her pulse was uniformly accelerated, seldom less than 100 in the minute.

October 29th.—In consequence of being a little irritated, she has severe pain in the head, with throbbing of the arteries; her pulse is 100, and beats with considerable force.—Mittatur sanguis e brachio ad uncias viij. vel x. The blood presented an exceedingly buffy and cupped appearance; the coagulum was so firm, that I could with difficulty pass my finger through it. This apparently trifling quantity of blood relieved the head greatly, but left her in such a state of syncope, that for many hours she could scarcely move hand or foot. Hyoscyamus was given to the extent of fifteen grains of the extract every four hours, with an occasional laxative. She slept much, but never waked refreshed, and there was uniformly a sensation of heaviness over the eyebrows.

On the 2d of November (the ninth day after parturition, reckoning that of delivery as the first), in consequence of a trifling domestic occurrence, she was thrown into a state of excessive irritability, and the symptoms of cerebral disorder were much increased: she complained greatly of a feeling of constriction within the head, as if the cranium was too small for the brain, and it was on that account compressed. The senses of sight, hearing, smell, and taste, were painfully acute; the pulse about 100. Eight leeches were applied to the temples, and a laxative administered.—In the evening, finding her no better, I requested a consultation, and Dr. Blundell was sent for, when it was agreed to apply six more leeches to the temple, to give her the digitalis in operative quantities; to have the hair removed, and keep the head constantly wet and cold with a lotion composed of equal parts of spiritus eth. sulph. and water.

November 3d (tenth day).—Somewhat, though not very materially, relieved; pulse still at 100.—Repetatur remedia.

Nov. 4th (eleventh day).—The feeling of oppression still continuing, eight leeches were again applied to the temple.—Continuatur cetera.

Nov. 5th (twelfth day).—Was called to her about two o'clock this morning, and found the symptoms very materially aggravated: she had a weak, small pulse of 124; a whitish, but somewhat moist, tongue; a very pallid countenance; subsultus of the muscles of the lower extremity, and a quivering also of those of the lower lip; with that peculiar, faint, earthy smell of the breath I have more than once noticed in moribund patients. It was now very evident that, although action was increasing, that power was rapidly on the decline. I again requested the assistance of Dr. Blundell, and in consultation it was agreed to put her fully under the influence of opium, and to continue the evaporating lotion freely. This medicine was ordered in doses of three grains of the extract every hour, till sleep was procured. She began about six o'clock in the morning, and at one in the afternoon I gave her the sixth dose, (making eighteen grains of ext. opii in seven hours.) The pulse was now (one o'clock,) down to ninety-two, full, and round, very different to the irritable pulse of the morning, and she appeared in a very tranquil state. Another three-grain dose of the opium was given her at five o'clock, and a draught, composed of liq. am. acet. et mist. camph., every four hours. She slept all the night, and nearly the whole of the following day: her breathing was stertorous during the night, but she could be very readily roused. She wandered a little, but not much, during her sleep, and awoke very much refreshed.

Nov. 6th.—Seemed considerably relieved this morning.—Repetatur haustus liq. am. acet.; capiat pil. opii hora somni. This pill was twice repeated, as she seemed rather restless, which at length procured her some refreshing sleep.

Nov. 7th.—Still on the mend, although she has had some slightly hysterical paroxysms, which distressed her greatly for the time. A little cascarilla infusion was now added to her draught.—Continuatur pil. opii nocte.

Nov. 8th.—Convalescent: took two pills last night, which procured her sleep. From this time she has had no relapse, and is gradually, though slowly, regaining her strength. The secretion of milk was never entirely checked, nor was the lochial discharge.

The foregoing case shows, in a striking manner, the utility of putting the system fully under the influence of opium, in this state of excessive irritability. Antiphlogistic medicines were had recourse to, with little or no benefit; but, so soon as this medicine began fairly to act, we find the pulse running down from 124 to 92: indeed, I think it very evident that we must have lost our patient but for this decided plan of treatment, as every thing seemed to indicate approaching dissolution.

My chief reason for publishing the case, is to excite the attention of my professional brethren to a form of disease which they must have every now and then met with, and which may be termed puerperal irritability, which is not noticed by authors, nor, as far as I am aware of, by our obstetric teachers, and which, consequently, is an exceedingly puzzling complaint to a young practitioner.

111, *Aldersgate-street; Nov. 23d*, 1824.

